# Results at the 1-Year Follow-Up of a Prospective Cohort Study with Short, Zirconia Implants

**DOI:** 10.3390/ma15165584

**Published:** 2022-08-15

**Authors:** Marc Balmer, Carolin Fischer, Miha Pirc, Christoph H. F. Hämmerle, Ronald E. Jung

**Affiliations:** Clinic of Reconstructive Dentistry, Center of Dental Medicine, University of Zurich, 8032 Zurich, Switzerland

**Keywords:** zirconia implant, dental implantology, osseointegration, ceramic, clinical, radiographic bone level

## Abstract

The objective of this study was to clinically and radiologically evaluate the performance of a short (8 mm), 1-piece, zirconia implant after an observation period of 1 year in function. A total of 47 patients with 1 missing tooth in the position of a premolar or molar were recruited. Short (8 mm), 1-piece, zirconia implants were placed and loaded after a healing period of 2 to 4 months with monolithic crowns made of 3 different materials. Implants were followed up for one year and clinically and radiologically assessed. A total of 46 implants were placed. One was excluded since no primary stability was achieved at implant placement. At the 1-year follow-up, mean marginal bone loss 1 year after loading was 0.05 ± 0.47 mm. None of the implants showed marginal bone loss greater than 1 mm or clinical signs of peri-implantitis. A total of 2 implants were lost during the healing phase and another after loading, resulting in a survival rate of 93% after 1 year. All lost implants showed a sudden increased mobility with no previous signs of marginal bone loss or peri-implant infection. The short, 8 mm, zirconia implants showed stable marginal bone levels over the short observation period of 1 year. Although they revealed slightly lower survival rates, they can be suggested for the use in sites with reduced vertical bone. Scientific data are very limited, and long-term data are not yet available, and therefore, they are needed.

## 1. Introduction

The clinical evidence for zirconia implants is currently limited to short-term observation periods. In short periods, zirconia implants show promising results in terms of a high survival rate and low marginal bone loss (MBL), especially for commercially available implant systems [1]. Furthermore, clinical studies on ceramic implants with an observation period of 5 or more years can confirm positive mid-term performance with survival rates of 94.3–100% [2,3]. Zirconia implants, therefore, may be considered as abutments for single crowns and fixed dental prostheses [4]. However, only a limited number of studies are available in the current literature, and long-term data are still missing.

Besides this limited amount of data, there is currently also no prospective clinical study that has specifically investigated the outcome of short or reduced-diameter zirconia implants. In various published studies on zirconia implants, the diameter and length of the implants were adapted to the individual clinical situation [2,5,6,7]. Due to the heterogeneous datasets of these studies, no conclusion can be drawn as to whether short or reduced-diameter ceramic implants show the same clinical behavior as longer or wider implants.

Short implants are defined as those measuring 8 mm or less in infra-bony length [8]. For titanium implants, various studies have shown that the survival rate of short implants does not differ significantly from that of longer implants [9,10,11]. In particular, if a vertical ridge augmentation is required for the placement of a longer implant, the placement of a short implant reduces the complexity of the surgical procedure, morbidity, and cost [12,13,14]. However, in contrast, there are also studies that report a tendency towards higher failure rates in short implants [15,16].

The evidence based on studies with titanium implants cannot be transferred directly to zirconia implants. Zirconia implants show different material properties and different surface topographies [17]. As a result, they may exhibit different clinical behaviors. Based on the results of our previous study [2], there is, at least, very limited evidence of the performance of short, 8 mm, 1-piece, zirconia implants. In that study, 6 implants with a diameter of 4 mm and a length of 8 mm were placed, serving as abutments for single-tooth restorations or fixed dental prostheses. All of the implants survived for up to 10 years to date.

Since there is currently no study specifically targeting the reliability of short, zirconia implants, the present study is intended to clinically and radiologically evaluate the performance of a 1-piece, zirconia implant with the shortest available length (8 mm) and with the narrowest diameter (4 mm) of the implant system, as investigated after an observation period of 1 year in function.

## 2. Materials and Methods

### 2.1. Study Design

This study was designed as a prospective, single-arm clinical trial with an observation period of 5 years. It was conducted in one university center (the Clinic of Reconstructive Dentistry, Center of Dental Medicine, University of Zurich, Zurich, Switzerland). Prior to inception, the study was registered with the Swiss Federal Complementary Database and with the international trial registry, the “German Clinical Trials Register” (DRKS00011146). Approval from the appropriate, constituted Competent Ethics Committee was obtained. The study was conducted in compliance with the investigation plan, the current version of the Declaration of Helsinki, and ISO EN 14155 [18], as well as with all national legal and regulatory requirements.

### 2.2. Participants

Patients with one missing tooth in the mandible or maxilla, in the position of a premolar or molar in need of an implant-supported, single-tooth restoration, were recruited. They had to be at least 20 years old, and they could not have any general medical contraindications for implant surgery. In the mandible, the implant site was required to have at least 10 mm of vertical bone height for the placement of an 8 mm implant with a 2 mm safety distance to the inferior alveolar nerve. In the maxilla, an area of at least 7 mm of vertical bone height was required, allowing for a crestal sinus elevation procedure [19] of a maximum of 1 mm with a deproteinized bovine bone material (BioOss^®^ Collagen, Geistlich Pharma, Wolhusen, Switzerland) if needed.

Heavy smokers (those who smoked more than 15 cigarettes per day), women who were pregnant or breastfeeding, persons with known addictive or non-compliant behavior, and those with a dependency relationship to the persons involved in the study were not included. Poor oral hygiene after the hygienic phase (a plaque index over 30%) and active periodontal disease prevented inclusion in the study.

Upon fulfilling all of the inclusion criteria and signing the informed consent, the patients were included in the study.

### 2.3. Intervention

All patients received a 1-piece, zirconia implant (VITA Zahnfabrik, Bad Säckingen, Germany) with an endosseous length of 8 mm and a diameter of 4 mm, which corresponds to the shortest and narrowest available implant in the applied implant system. The implant surface characteristics (cer.face14, ceramic.implant, VITA Zahnfabrik) have been previously described in detail [20]. In brief, the endosseous surface has a moderately rough surface, which is sandblasted and subsequently acid-etched, while the transmucosal part is polished. After surface treatment, the implant is heat-treated to recover the tetragonal phase of zirconia. For the cementation of the crown, the implant provides an integrated 4.5-mm tall abutment.

**Figure 1:**
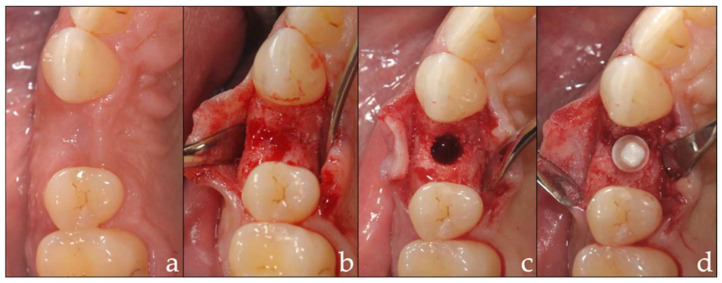
(**a**–**d**) Implant surgery: (**a**) initial situation; (**b**) flap elevation; (**c**) drilling procedure; (**d**) implantation.

The implants were inserted under local anesthesia (Ubistesin forte, 3M, Saint Paul, MN, USA) after the elevation of a mucoperiosteal flap. The position of the implant was defined according to the future prosthetic restoration. The insertion depth was adjusted to the level of the prospective crown margin in order to achieve an adequate emergence profile (Figure 1a–d). If a bony defect occurred during implant placement (exposure of the endosseous implant surface), a guided bone regeneration with a deproteinized bovine bone material (BioOss^®^ Spongiosa Granules, Geistlich Pharma) and a collagen membrane (BioGide^®^ Membrane, Geistlich Pharma) was carried out.

**Figure 2:**
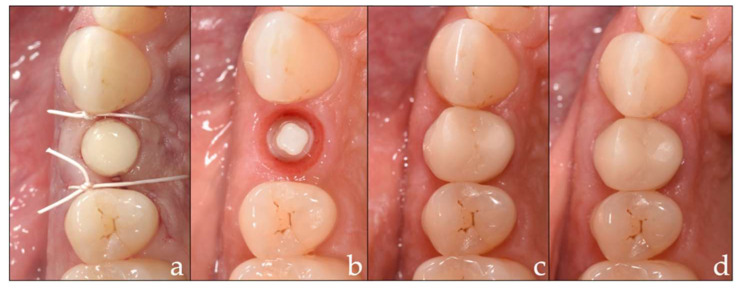
(**a**–**d**) Implant restoration: (**a**) prefabricated zirconia cap situation; (**b**) final impression; (**c**) baseline examination; (**d**) 1-year follow-up.

All implants underwent transmucosal healing (one-stage procedure). A prefabricated zirconia cap without occlusal and approximal contacts was placed with a temporary cement (Freegenol, GC Corporation, Tokyo, Japan) to cover the shoulder of the implant so as to retain the mucosa (Figure 2a). Patients were instructed to rinse with a 0.2% chlorhexidine rinsing solution for 1 min twice a day until the next visit. Patients were prescribed analgesics for individual pain relief (400 mg ibuprofen) and in the case of bone augmentation, antibiotics were additionally dispensed (co-amoxicillin 625 mg, 3 times a day for 1 week). The sutures were removed 1 week postoperatively.

### 2.4. Prosthetic Insertion and Follow-Up

One month after implantation, a reexamination was performed. At this point in time, the implants were randomly assigned to one of the following three groups:

1. Restoration with monolithic zirconia (VITA YZ^®^, VITA Zahnfabrik)
2. Restoration with monolithic polymer-infiltrated ceramic (VITA ENAMIC^®^, VITA Zahnfabrik)
3. Restoration with monolithic polymer (VITA CAD-Temp^®^, VITA Zahnfabrik).

Final impressions were taken 2 to 4 months after implantation with a polyether impression material (Permadyne^TM^, 3M, Saint Paul, MN, USA) using an implant pick-up and the closed-tray technique (Figure 2b). Final crowns were cemented after close examination of the biological, functional, and aesthetic aspects. (For the zirconia, Rely X Unicem 2 Automix (3M) was used; for the polymer-infiltrated ceramic, VITA Adiva F-Cem (VITA Zahnfabrik) was used; and for the polymer, Rely X Ultimate (3M) was used).

The baseline examination was performed 1–2 weeks after crown cementation (Figure 2c), and the first follow-up took place after 1 year (Figure 2d). Further follow-up examinations are scheduled to take place 3 and 5 years post-implantation.

### 2.5. Examinations and Analyses

**Figure 3:**
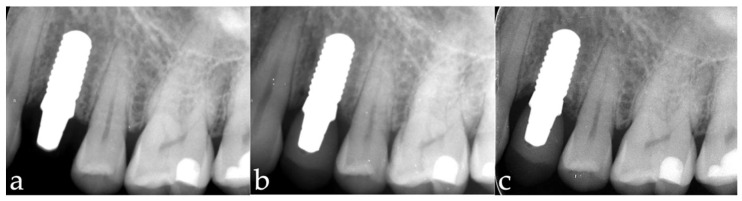
(**a**–**c**) Radiographic series: (**a**) after implantation; (**b**) crown delivery; (**c**) 1-year follow-up-up.

The primary objective of this ongoing study is to evaluate the vertical change of the mean marginal bone level over an observation period of 5 years after loading with the final crown. In the interim stage, data were collected one year after crown placement. Standardized periapical radiographs were taken upon implant placement (Figure 3a), crown delivery (Figure 3b), and at the 1-year follow-up (Figure 3c) with the parallel technique. Radiographs were imported into an open-source software (ImageJ, National Institutes of Health, Bethesda, MD, USA) and magnified tenfold. The images were calibrated using the known distance of four implant thread pitches. The distances between the implant shoulder and the first-bone-to-implant contact were measured at the mesial and distal sites to evaluate the vertical marginal bone level. An increase of this distance over time is considered to be marginal bone loss (MBL), and a decrease as marginal bone gain.

The secondary objectives are to evaluate the implant survival, clinical parameters, and biological complications (mucositis, peri-implantitis). If present, the extent of buccal osseous dehiscence at implantation was measured. Furthermore, the thickness of the mucosa was measured buccally 1 mm below the gingival margin with an endodontic file at re-examination. The stability of the implant was clinically examined at every appointment, starting with the appointment for the impression for the final restoration. The presence of plaque, bleeding on probing (BoP), and probing pocket depth (PD) were measured with a periodontal probe at 6 sites around each implant at baseline and again at the 1-year follow-up. The width of the keratinized mucosa was measured centrally, buccally of the implant.

The tertiary outcomes are the functional and esthetic outcomes of the three different restoration materials which differ in their elastic modulus. These prosthetic-level results will be reported in detail in a separate publication.

### 2.6. Statistical Analysis

A power analysis was carried out to determine the sample size (45 patients). If a patient dropped out during the active phase of treatment, the recruiting of patients was continued in order to replace the lost patients until the calculated sample size of loaded implants was achieved.

Data were calculated in Excel (Microsoft Corp., Redmond, WA, USA), and all statistical analyses and plots were computed with the statistical software R [21], including the package tidyverse [22]. The primary objective (mean MBL) was tested with the *t*-test or Wilcoxon test in the case that distributional assumption was not met. Secondary endpoints were reported descriptively using means and standard deviations. The survival of the object was plotted to obtain the Kaplan–Meier plot, including 95% confidence intervals. The level of significance was set at 0.05 for all analyses.

## 3. Results

A total of 47 patients (34 female and 13 male) (age at inclusion: 55.7 ± 14 years (mean ± SD)) were included in the study. In one patient, the primary stability of the study implant could not be achieved. Consequently, a wider implant was placed, which led to that patient’s exclusion from the study. The remaining 46 implants were placed according to the study protocol. The distribution of the implants, incidence of buccal osseous dehiscence after implant placement, and randomized allocation of the different crown materials are summarized in Table 1. In 18 of the cases (39%), dehiscence was observed after the placing of the implant. The mean defect height was 1.3 ± 2.2 mm, defect width was 1.0 ± 1.8 mm, and defect depth was 0.6 ± 1.2 mm. At re-examination, 43 ± 16 days after implantation, a mean thickness of 2.5 ± 0.8 mm for the buccal mucosa was measured.

A total of 2 implants lost their osseointegration 5 months after implantation without any signs of infection and had to be removed before the final restoration. Another implant was no longer stable after 4 months post-implantation. During a try-in of the restoration, the implant moved slightly. This implant was left in place, not loaded for another 6 months, and successfully restored 1 year after implant placement. A total of 44 implants were restored after 155 ± 63 days with monolithic crowns (15 zirconia, 14 polymer-infiltrated ceramic, and 15 polymer). Between baseline and the 1-year follow-up, another implant (FDI 24) was lost eight months after implantation and three months after loading (polymer crown). The patient noticed a sudden mobility of the implant. The implant did not show increased marginal bone loss, increased probing depth or more bleeding on probing, but was however no longer integrated.

A total of 42 patients were eligible for the 1-year follow-up (386 ± 44 days after baseline). One patient refused to attend the follow-up during the COVID-19 pandemic but confirmed the survival of the implant. In two patients, clinical parameters could be evaluated, but no radiograph taken.

### 3.1. Marginal Bone-Level Changes

The implants were placed at a mean depth so that the transition line between the smooth, transmucosal surface and the rough, endosseous surface was located, on average, 1.24 ± 0.57 mm subcrestally. A mean MBL of 0.67 ± 0.59 mm could be observed between implantation and loading.

**Figure 4:**
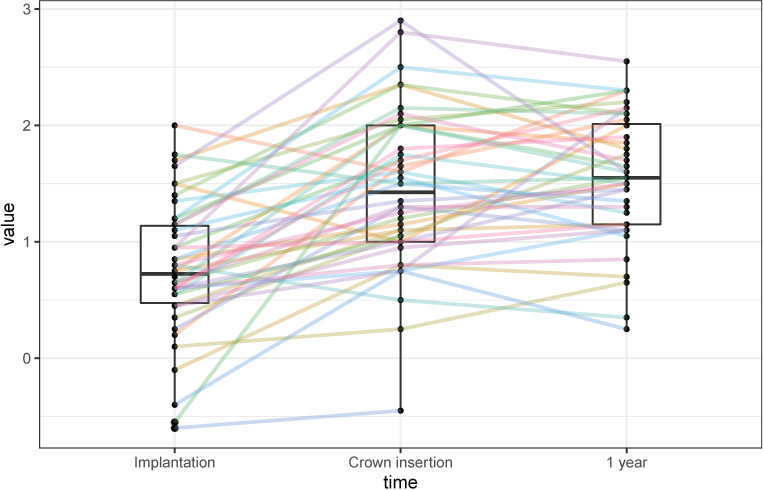
Individual marginal bone-level changes over time (values in mm).

The mean MBL between loading and the 1-year follow-up was 0.05 ± 0.47 mm. The individual changes are shown in Figure 4, in which the majority follows a common pattern. A total of 35 implants (87.5%) showed a bone gain or an MBL of less than 0.5 mm. A total of 5 implants (12.5%) lost between 0.5 and 1.0 mm, and none of the implants exceeded 1.0 mm after loading. Analyses showed that neither the material composition of the restoration (*p* = 0.5, 0.6, 0.8), the jaw (maxillary/mandibular: *p* = 0.8), the tooth type (molar/premolar: *p* = 0.9), nor the extent of dehiscence after implantation (*p* = 0.8) had a statistically significant influence on the MBL after loading.

A mean vertical bone loss of 0.1 ± 0.49 mm was measured, distal from the mesial neighboring tooth and mesial from the distal neighboring tooth, in the observation period between implantation and loading, and a loss of 0.14 ± 0.48 mm was measured after loading.

### 3.2. Implant Survival

**Figure 5:**
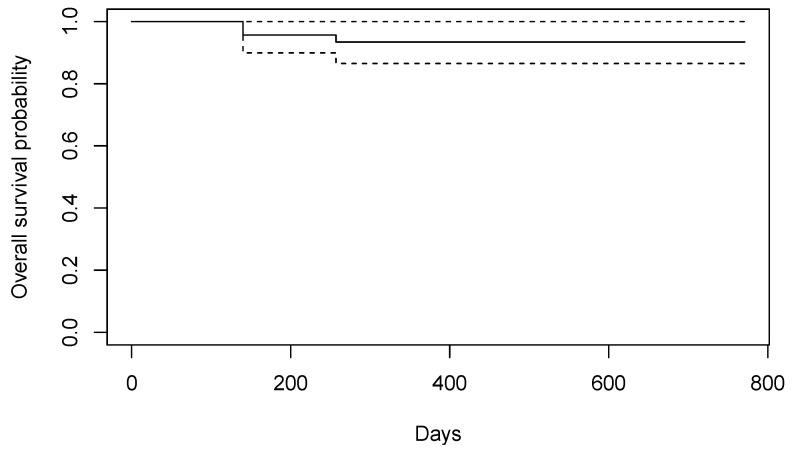
Kaplan–Meier survival plot, including the 95% confidence intervals.

A total of 46 implants were placed. Two implants were lost before loading, and one was lost after loading. One patient refused to return for the 1-year follow-up but confirmed implant survival. The survival rate after 1 year in function was 93%. The Kaplan–Meier plot, including the 95% confidence intervals, is shown in Figure 5.

### 3.3. Clinical Parameters

Clinical parameters, such as the presence of plaque, BoP, mean probing depth, and mean width of keratinized mucosa, are listed in Table 2. The mean probing depth around the implants at the 1-year follow-up was 3.4 mm, and none of the implants had a probing depth greater than 4 mm.

## 4. Discussion

In this ongoing, prospective clinical trial, short, 8 mm, zirconia implants were placed, restored with monolithic crowns composed of one of three different materials, and followed-up 1 year after loading.

This is the first publication of a study evaluating the clinical performance of short, zirconia implants. The definition of a “short implant” is not entirely consistent and has changed over the years. While an older review considered implants with a length ≤ 10 mm to be short [23], a more recently published review only included implants ≤ 6 mm [24]. In the present study, implants were applied with the shortest length (8 mm) and the narrowest diameter (4 mm) available from the implant system used. Thus, the implants had the smallest possible endosseous surface, making the analysis of their clinical performance highly interesting.

In the current investigation, marginal bone loss in the first year after loading amounted to 0.05 mm. Marginal bone-level changes of ≤0.5 mm should be interpreted cautiously due to the consideration of errors in the measurement of bone levels [25]. However, the value of MBL is in line with other studies of one-piece zirconia implants [7,26,27]. Of particular interest is the comparison of our study with one in which the same zirconia implant system was investigated under a similar study design [2,28,29] although not all of the inclusion criteria are consistent between the two studies (including the threshold for heavy smokers and the oral hygiene stipulations). In contrast with the current investigation, implants of different lengths were used, which were, on average, longer (mean length: 10.3 mm). That study also showed an overall stable vertical marginal bone level over the first year after loading. On the other hand, it also showed that, within the study, implant length had no significant influence on MBL. This conclusion had already been reached in several randomized studies with titanium implants that compared short implants to standard-length implants [15,30,31]. However, a limitation of the current study is the absence of a comparison group in terms of short, titanium implants. Therefore, we cannot conclude whether short, ceramic implants can be recommended as an alternative to titanium implants in sites with limited vertical bone height.

The fact that the means and the majorities of the individual values in the current investigation did not exceed the threshold of 0.5 mm [25] indicates a stable marginal bone level without clinically significant changes for short, zirconia implants in the short observation period of 1 year after loading.

During the observation period between implantation and crown insertion, the bone level changed by 0.67 ± 0.59 mm. In our previous study, a similar value of 0.78 ± 0.79 mm was observed [2]. This may be attributed to a process of physiological remodeling after implant placement. The amount of remodeling is influenced by a number of local and systemic factors, particularly the position of the transition line between the rough and smooth surfaces and the implant geometry [32]. In the current study, this transition line was indeed 1.2 mm subcrestally positioned and may have been a reason for the resorption before loading. This observation has already been discussed in detail for the studied implant system (ceramic.implant, VITA Zahnfabrik) [20] and was also observed in studies with other one-piece, zirconia implants (PURE, Straumann, Basel, Switzerland) [27,33] (Ziraldent FR1, Metoxit AG, Thayngen, Switzerland) [7]. For this reason, a healthy peri-implant situation is only characterized by the absence of bone loss beyond the crestal bone-level changes resulting from initial bone remodeling [25].

In the present study, two implants were lost during the healing period before loading, and one was lost after loading, resulting in a survival rate of 93%. This rate is slightly inferior to the published survival rates of zirconia implants, according to an analysis of recent, systematic reviews, of 95.6% [34] and 98.3% [1]. In the above-mentioned study with identical, but longer, zirconia implants [29], the survival rate after 1 year was also higher at 98.3%. For titanium implants, data showed lower survival rates for short implants, as derived from randomized clinical studies, in which the performance of short and standard-length implants was investigated in situations where a standard-length implant could be placed without vertical bone augmentation. After an observation period of 5 years, the survival rates for short and standard-length, non-splinted, titanium implants ranged between 87–91% and 97–100%, respectively [15,16]. In the present study, the implants had a combination of a short length with the narrowest possible diameter and were exposed to potential forces in the oral cavity during the healing phase due to the one-piece design with an integrated abutment. In comparison with a longer, one-piece implant or with a two-piece implant combined with a two-stage procedure, these could be considered as additional risk factors for early implant loss.

All of the three implants were lost after a sudden onset of mobility without any signs of inflammation. In a 6-year prospective clinical study of 2-piece, ceramic implants, Cionca et al. also reported that, out of 6 implants lost, 1 failed to osseointegrate, and 5 implants were affected by a sudden loss of stability without signs of inflammation at the first year after loading [35]. The authors described this phenomenon as so-called “aseptic loosening”, which is typically limited to the first year and could be explained by disintegration or a premature loading. It also has been discussed in a randomized clinical trial with short, titanium implants [15]. The authors discussed mechanical microfractures as a possibility that led to the loss of the previously achieved osseointegration in short implants.

However, at this point in time, it must be stated that the reason for the implant failures cannot be determined conclusively.

None of the implants showed marginal bone loss greater than 1 mm or a probing depth deeper than 4 mm after the first year. Thus, it can be concluded that none of the implants were affected by peri-implantitis after 1 year.

## 5. Conclusions

Short, 8 mm, zirconia implants showed stable marginal bone levels over a short observation period of 1 year. Although they revealed slightly lower survival rates than standard-length, zirconia implants, they can be suggested for use in sites with reduced vertical bone. Scientific data are very limited, and long-term data are not yet available, and therefore, they are needed.

## Figures and Tables

**Table 1:** Distribution of implants (position according to World Dental Federation notation (FDI)), incidence of buccal osseous dehiscence after implant placement, and randomized allocation of the different crown materials.

	Implant Position (FDI)							
	17	16	15	14	24	25	26	27
n implants (proportion of total implants)	0 (0%)	2 (4%)	5 (11%)	4 (9%)	7 (15%)	4 (9%)	1 (2%)	0 (0%)
n implants with dehiscence (proportion at each position)		0 (0%)	2 (40%)	2 (50%)	3 (43%)	2 (50%)	1 (100%)	
Allocation of crown material n(Z/C/P) *		0/0/2	2/0/3	1/3/0	1/1/5	2/2/0	1/0/0	
	**Implant Position (FDI)**							
	**47**	**46**	**45**	**44**	**34**	**35**	**36**	**37**
n implants (proportion of total implants)	0 (0%)	10 (22%)	4 (9%)	2 (4%)	0 (0%)	0 (0%)	7 (15%)	0 (0%)
n implants with dehiscence (proportion at each position)		3 (30%)	4 (100%)	1 (50%)			2 (29%)	
Allocation of crown material n(Z/C/P) *		3/4/3	2/1/1	0/2/0			3/2/2	

* Z: zirconia, C: polymer-infiltrated ceramic, P: polymer.

**Table 2:** Clinical parameters (mean percentage of measured sites with presence of plaque (PI), mean probing depth (PD), mean percentage of measured sites with bleeding on probing (BoP), and mean width of the keratinized mucosa (Kerat. T.)) per implant and neighboring teeth at baseline and at 1-year follow-up.

	Implants				Neighboring Teeth			
	PI	PD	BoP	Kerat. T.	PI	PD	BoP	Kerat. T.
Baseline	14 ± 25%	3.3 ± 0.4 mm	11 ± 22%	3.3 ± 1.5 mm	13 ± 17%	2.6 ± 0.3 mm	8 ± 10%	3.5 ± 1.4 mm
1 y Follow-up	22 ± 20%	3.4 ± 0.5 mm	16 ± 16%	3.4 ± 1.5 mm	15 ± 15%	2.6 ± 0.5 mm	11 ± 11%	3.5 ± 1.3 mm

## Data Availability

The data presented in this study are available on request from the corresponding author. The data are not publicly available due to ethical reasons.
